# Association between glycosylated hemoglobin levels, diabetes duration, and left ventricular diastolic dysfunction in patients with type 2 diabetes and preserved ejection fraction: a cross-sectional study

**DOI:** 10.3389/fendo.2023.1326891

**Published:** 2023-12-20

**Authors:** Na Li, Mengnan Zhao, Lingling Yuan, Yanxia Chen, Hong Zhou

**Affiliations:** Department of Endocrinology, the Second Hospital of Hebei Medical University, Shijiazhuang, Hebei, China

**Keywords:** glycated hemoglobin, diabetic duration, type 2 diabetes mellitus, ventricular diastolic dysfunction, risk factors

## Abstract

**Background:**

We aimed to explore the intricate interplay between glycated hemoglobin (HbA1C) levels, disease duration, and left ventricular diastolic dysfunction in patients with type 2 diabetes mellitus (T2DM) characterized by preserved ejection fraction.

**Methods:**

A cross-sectional study was conducted at the Second Affiliated Hospital of Hebei Medical University from January 2022 to December 2022. A total of 114 inpatients from the Department of Endocrinology were randomly selected based on the inclusion and exclusion criteria. Patients with T2DM were stratified into three subgroups, each comprising 38 patients, based on disease duration and HbA1C levels. A sub-analysis was conducted to explore variations among these three distinct groups. A control group comprised 38 age, gender, body mass index (BMI), and smoking habit-matched healthy volunteers form the Physical Examination Center of the same hospital. General demographic information, biochemical results, and echocardiographic data were collected, and correlation and linear regression analyses were performed.

**Results:**

Diabetic patients exhibited lower E/A values (0.85 (0.72, 1.17) vs. 1.20 (0.97, 1.30)) and elevated E/e’ values (9.50 (8.75, 11.00) vs. 9.00 (7.67, 9.85)) compared to their normal controls. In the subgroup analysis, patients with a disease duration exceeding 2 years displayed reduced E/A values (0.85 (0.75, 1.10) vs. 1.10 (0.80, 1.30)) and elevated E/e’ values (9.80 (9.20, 10.80) vs. 8.95 (7.77, 9.50)) in comparison to those with a disease duration of ≤2 years, p<0.05. Among patients with a disease duration surpassing 2 years, those with higher HbA1C levels exhibited lower E/A values (0.80 (0.70, 0.90) vs. (0.85 (0.75, 1.10)) and higher E/e’ values (11.00 (9.87, 12.15) vs. 9.80 (9.20, 10.80)) in contrast to patients with low HbA1C levels, p<0.05. Multiple linear regression analysis identified HbA1C (β=0.294, p<0.001) and disease duration (β=0.319, p<0.001) as independent risk factors for the E/A value in diabetes patients. Furthermore, HbA1C (β=0.178, p=0.015) and disease duration (β=0.529, p<0.001) emerged as independent risk factors for the E/e’ value in diabetic patients.

**Conclusions:**

In individuals with T2DM exhibiting preserved ejection fraction, the presence of left ventricular diastolic dysfunction is significantly associated with HbA1C levels and the duration of diabetes.

## Introduction

Diabetes stands as an independent risk factor for both left ventricular hypertrophy and congestive heart failure (HF), with diabetic patients experiencing a more adverse prognosis in heart failure compared to their non-diabetic counterparts (1). The trajectory of diabetic cardiomyopathy (DCM) unfolds as diabetes initiates diastolic dysfunction, progresses to systolic dysfunction, and culminates in refractory HF, unrelated to hypertension, coronary heart disease, or valvular disease. Numerous studies affirm that diabetes not only alters the structure and function of the myocardium bot also heightens the risk of cardiovascular events for affected individuals (2–4). In its early stages, DCM may manifest as a symptomatic diastolic dysfunction with preserved ejection fraction (EF), revealing itself through subtle left ventricular stiffness and cardiac hypertrophy (5, 6). Typically, the initial signs of diabetic left ventricular diastolic dysfunction include delayed left ventricular filling and diastole, often unaccompanied by impaired cardiac systolic function (7). Studies report that the incidence of left ventricular diastolic dysfunction in patients with type 2 diabetes mellitus (T2DM) ranges from 35% to 60% (8–10). Despite this prevalence, diastolic function is not routinely assessed in clinical practice, even though it represents an early feature of DCM (11). Cardiac complications in patients with diabetes often raise concerns only when overt symptomatic heart failure manifests. While cardiac magnetic resonance imaging (CMRI) is considered the gold standard for assessing ventricular diastolic function (1, 12), its use remains limited in clinical settings. Recent advancements in echocardiography technology position it as the primary choice for most clinical practices. Current guidelines from the American Society of Cardiac Ultrasound and the European Society of Cardiovascular Imaging emphasize that Doppler flow imaging parameters can effectively assess diastolic function (7). Notably, several factors, including higher HbA1C levels, are associated with an increased risk of worsening diastolic function in DM2 patients (13, 14). However, conflicting results emerge from studies assessing whether intensive glycemic control significantly reduces the risk of cardiovascular events and heart failure in patients with diabetes (15). This discrepancy underscores the need for further investigation into the control of HbA1C levels and its potential impact on left ventricular diastolic function and its relationship with DCM. Therefore, our study aimed to elucidate the intricate association between glycated hemoglobin levels, diabetes duration, and left ventricular diastolic dysfunction in patients with T2DM and preserved ejection fraction. The anticipated findings aspire to enhance the monitoring and evaluation of cardiac function, enabling the early detection of cardiovascular complications in individuals with T2DM.

## Methods

A cross-sectional study was conducted at the Second Affiliated Hospital of Hebei Medical University from January 2022 to December 2022, designed and implemented in strict adherence to the Declaration of Helsinki and International Ethical Guidelines for Biomedical Studies Involving Human Subjects. The protocol received approval from the Ethics Committee of the Second Affiliated Hospital of Hebei Medical University. In this study, patient consent was not required as it was approved under a waiver.

### Patients and grouping

A total of 114 hospitalized patients diagnosed with T2DM at the Department of Endocrinology of the Second Affiliated Hospital of Hebei Medical University were included and avoiding key exclusion criteria (16). Exclusion criteria comprised: (1) Previous or current diagnoses of hypertension, coronary heart disease, rheumatic heart disease, or other heart conditions; (2) Occurrence of diabetic ketoacidosis or hyperosmolar coma within the past 3 months; (3) Severe liver dysfunction, with transaminase levels 2.5 times higher than the normal value;(4) Urinary Albumin-to-creatinine ratio (ACR) ≥ 300mg/g or/and Estimated Glomerular Filtration Rate (eGFR)≤ 60ml/min/1.73m^2^ (5) Left ventricular ejection fraction (LVEF)<50%.

Participants were grouped based on disease duration and HbA1C levels: patients with a disease duration of ≤2 years (initial diabetes, ID group); patients with a disease duration of >2 years and HbA1C>7.5% (higher-HbA1C diabetes, HD group); patients with a disease duration of >2 years and HbA1C ≤ 7.5% (lower-HbA1C diabetes, LD group). Additionally, 38 healthy volunteers undergoing simultaneous physical examinations at the same hospital served as normal controls. Age, gender, body mass index (BMI), smoking, and hypoglycemic drug usage were matched using a propensity score of 1:1 among these subgroups.

### Collected information

General information, including diabetes duration, smoking history, gender, age, BMI, medication usage, and systolic and diastolic pressures, was extracted from the electronic medical record system. Biochemical indicators, such as HbA1C, C-reactive protein (CPR), creatinine (Cr), total cholesterol (TC), triglycerides (TG), high-density lipoprotein cholesterol (HDL-C), uric acid (UA), and low-density lipoprotein cholesterol (LDL-C), were meticulously recorded.

### Echocardiography examination

The ultrasound instrument, ARIETTA 60 produced by Hitachi in Japan, with a probe frequency of 4.0 Hz, was used for the echocardiography assessment of patients included in the study. Each examination was performed three times. The LVEF was verified twice using two-dimensional M-mode ultrasound and the biplane Simpson method (17, 18). Left ventricular end- systolic volume (LVESV) and left ventricular end-diastolic volume (LVEDV) were measured at the left ventricular long- axis section. The E/A and E/e’ values were calculated based on the ratios of E peak (maximum early diastolic blood flow velocity of the mitral valve), A peak (maximum atrial systolic blood flow velocity), and e’ peak (early diastolic motion velocity of the mitral annulus).

### Statistical analysis

Statistical analysis was conducted using SPSS 26.0. The normality of continuous data was assessed using the Kolmogorov-Smirnov test. Normally distributed measurement data were presented as mean ± standard deviation.Single- factor analysis of variance was applied for comparisons among multiple groups, with LSD tests for inter-group comparisons. Non-parametric data were presented as median and interquartile range, and the Kruskal-Wallis test was employed for inter-group comparisons of nonparametric continuous variables. Counting data were expressed as the number of cases, and inter-group comparisons were conducted using the Chi-squared test. Correlation analyses for measurement and counting data were done using Pearson’s correlation for normally distributed variables and Spearman’s correlation for non-normally distributed variables. Multivariable linear regression analysis was performed to identify independent predictors of cardiac diastolic function. A significance level of P<0.05 was considered statistically significant, and all comparisons and correlations were two-tailed.

## Results

### Clinical characteristics in each group

**Figure 1:**
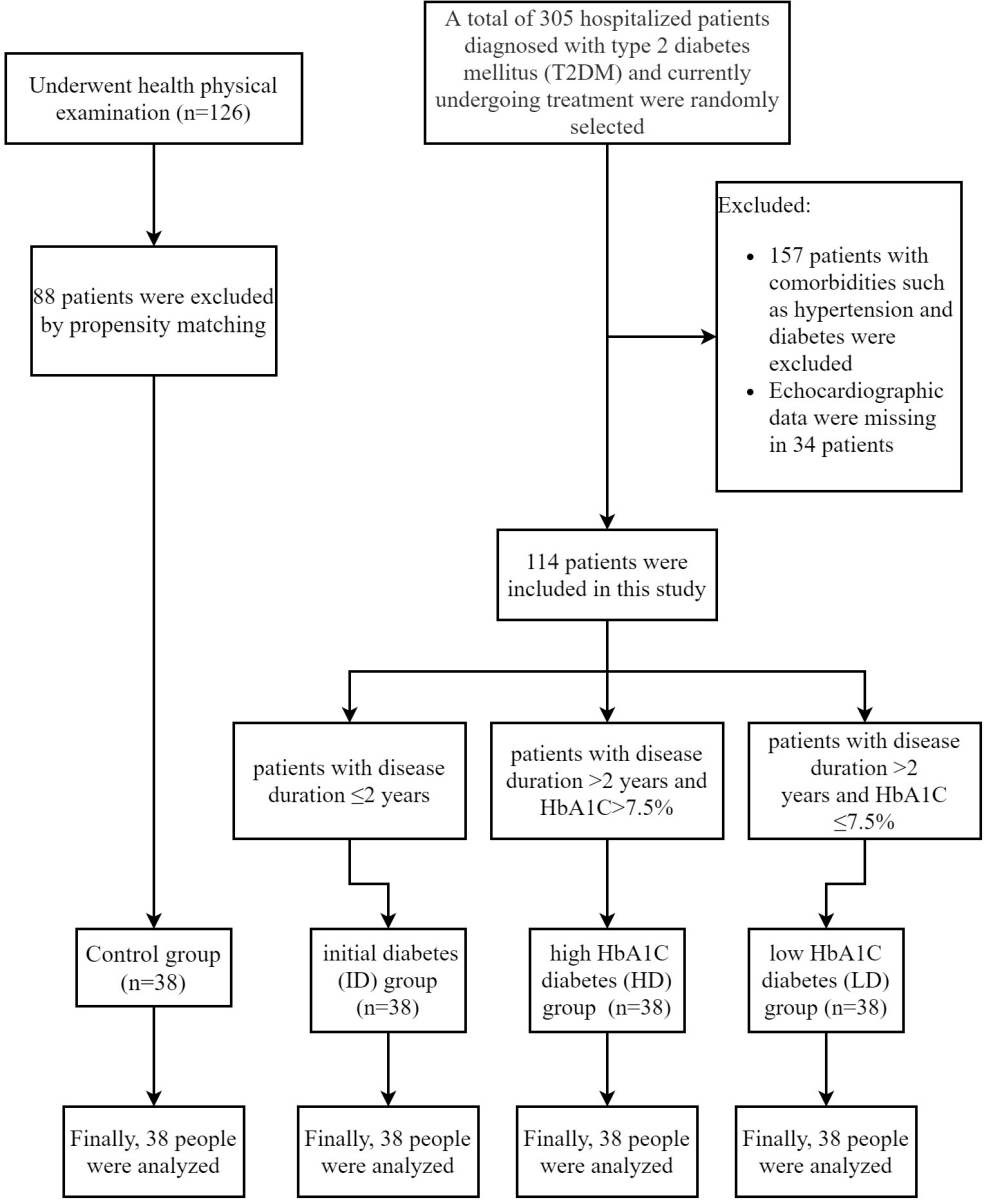
Flow chart of the inclusion process.

A total of 152 patients were enrolled and divided into the T2DM (n=114) and control groups (n=38) (**Figure 1**). Within the T2DM group, patients were further categorized into three subgroups: T2DM with a disease duration of ≤2 years (ID group, n=38); T2DM with a disease duration of >2 years and HbA1C>7.5% (HD group, n=38); patients with a disease duration of >2 years and HbA1C ≤ 7.5% (LD group, n=38). T2DM patients had significantly higher levels of TG, CRP, and HbA1C compared to controls (p< 0.05). Moreover, both ID and HD patients displayed elevated TG and HbA1C levels compared to controls and the LD group. There were no significant differences in CRP among the three subgroups within the T2DM population, as shown in **Table 1**.

**Table 1:** Clinical characteristics in each group.

Parameter	Cs (n=38)	DM (n=114)	ID (n=38)	LD (n=38)	HD (n=38)
Duration of disease (years)	–	5.00 (2.00, 10.00)	1.00 (0.00, 2.00)	7.00 (4.00, 11.00)	1.00 (6.50, 12.50)
Male [n (%)]	11 (28.94)	29 (25.43)	9 (23.68)	9 (23.68)	11 (28.94)
Age (years)	50.00(46.00. 54.50)	51.00 (47.50, 57.00)	51.5(43.00, 54.25)	51.00(48.00, 58.00)	51.5(48.00, 58.00)
BMI (kg/m^2^)	24.50(22.33, 27.02)	25.20 (23.35, 26.75)	25.39(23.34, 26.68)	25.30(24.00, 26.89)	25.00(22.92, 26.70)
SBP (mmHg)	118.71 ± 9.95	118.64 ± 10.67	117.16 ± 11.63	118.59 ± 8.74	120.05 ± 11.50
DBP (mmHg)	78.00 (70.00, 85.00)	78.00 (73.00, 83.00)	78.00 (70.50, 83.00)	81.00 (75.00, 85.25)	76.00 (72.50, 81.50)
Smoking [n (%)]	10 (26.31)	27 (23.68)	10 (26.31)	8 (21.05)	9 (23.68)
Medication [n (%)]					
Metformin	–	–	23 (60.52)	22 (57.89)	20 (52.63)
Insulin	–	–	8 (21.05)	9 (23.68)	9 (23.68)
SGLT2 inhibitor	–	–	2 (5.26)	3 (7.89)	3 (7.89)
GLP-1 RA	-	–	2 (5.26)	3 (7.89)	2 (5.26)
HbA1C (%)	5.40 (5.15, 5.70)	8.30 (7.30, 9.40)a	8.50 (7.97, 9.72) a	7.10 (6.60, 7.40) ab	9.40 (8.80, 9.62) ac
TG (mmol/L)	1.34 (0.97.1.69)	2.04 (1.47, 3.14)a	2.35 (1.64, 3.08) a	1.81 (1.29, 1.99) ab	2.35 (1.81, 3.17) ac
TC (mmol/L)	4.44 (4.25, 5.00)	4.34 (3.82, 4.86)	4.17 (3.50, 4.85)	4.36 (3.99, 4.64)	4.44 (3.84, 5.16)
HDL (mmol/L)	1.07 ± 0.16	1.03 ± 0.27	1.01 ± 0.26	1.09 ± 0.27	1.00 ± 0.26
LDL (mmol/L)	2.64 ± 0.69	2.64 ± 0.68	2.52 ± 0.73	2.72 ± 0.70	2.76 ± 0.90
UA (umol/L)	293.05 ± 93.52	299.59 ± 82.26	293.50 ± 77.35	312.51 ± 87.61	292.39 ± 81.93
Cr (mmol/L)	61.00 (55.00, 65.00)	58.00 (54.00,66.00)	61.00 (54.5, 67.00)	56.00 (54.00, 62.25)	57.00 (52.00, 66.50)
CRP (mg/L)	0.80 (0.60, 0.80)	2.90 (1.80, 4.30) a	2.30 (1.64, 3.08) a	2.61 (1.27, 3.73) a	3.40 (2.10, 4.55) a

BMI, body mass index; SBP, systolic blood pressure; DBP, diastolic blood pressure; SGLT2, Sodium-glucose cotransporter-2, GLP-1 RA, glucagon-like peptide-1 receptor agonist; HbA1C, glycated hemoglobin; CPR, C-reactive protein; Cr, creatinine; TC, total cholesterol; TG, triglycerides; HDL-C, high-density lipoprotein cholesterol; UA, uric acid; LDL-C, low-density lipoprotein cholesterol; Cs, Control group; DM, diabetes Mellitus; ID, initial diabetes group (patients with a disease duration of ≤2 years); HD, high HbA1C diabetes (HD) group (patients with a disease duration of >2 years and HbA1C>7.5%); LD, the low HbA1C diabetes (LD) group (patients with a disease duration of >2 years and HbA1C ≤7.5%).

a: Compared with Cs group, p<0.05.

b: Compared with ID group, p<0.05.

c: Compared with LD group, p<0.05.

### Echocardiography evaluation of cardiac systolic and diastolic function in each group

T2DM patients exhibited significantly higher E/e’ values and lower E/A index values compared to controls (p<0.05). Both the LD and HD groups displayed increased E/e’ values and decreased E/A index values compared to controls and the ID group (p<0.05). Notably, the HD group demonstrated a higher E/e’ and a lower E/A compared to the LD group. However, there were no significant differences between the T2DM and control groups in terms of LVEF values (65.66 ± 4.3 vs. 66.62 ± 4.51; p=0.25). Additionally, there were no significant differences in LVEF values among three subgroups within the T2DM patients (all p>0.05). Other functional parameters also showed no significant differences between T2DM patients and controls (**Table 2**).

**Table 2:** Cardiac systolic and diastolic functional parameters in each subject.

Indicator	Cs (38)	DM (114)	ID (38)	LD (38)	HD (38)
LVEF (%)	65.66 ± 4.30	66.62 ± 4.51	67.46 ± 4.52	66.29 ± 4.47	66.11 ± 4.54
E/A	1.20(0.97, 1.30)	0.85 (0.72, 1.17) a	1.10(0.80, 1.30)	0.85(0.75, 1.10) ab	0.80 (0.70, 0.90) abc
E/e’	9.00(7.67, 9.85)	9.50(8.75, 11.00) a	8.95(7.77, 9.50)	9.80(9.20, 10.80) ab	11.00(9.87, 12.15) abc
LVEDV (ml)	101.00(85.75, 123.00)	103.45(88.00, 118.50)	108.00(92.00, 123.00)	102.00(85.00, 115.00)	102.50(87.50, 130.25)
LVESV (ml)	35.00(29.00, 43.25)	36(31.50, 42.50)	35.50(31.50, 40.25)	37.00(31.00, 41.00)	36.00(30.75, 44.25)

LVEF, left ventricular ejection fraction; LVESV, left ventricular end- systolic volume; LVEDV, left ventricular end- diastolic volume; Cs, Control group; DM, diabetes Mellitus; ID, initial diabetes group (patients with a disease duration of ≤2 years); HD, high HbA1C diabetes (HD) group (patients with a disease duration of >2 years and HbA1C>7.5%); LD, the low HbA1C diabetes (LD) group (patients with a disease duration of >2 years and HbA1C ≤7.5%).

a: Compared to Cs group, p<0.05.

b: Compared to ID group, p<0.05.

c: Compared to LD group, p<0.05.

### Correlation analysis and multivariate regression analysis of E/A, E/e’, and other clinical indicators

The univariate correlation coefficients between E/A, E/e’ values, and clinical indexes in T2DM patients were summarized in **Table 3**.

**Table 3:** Correlation analysis of E/A, E/e’ and other clinical indicators.

Indicator	E/AR value	P value	E/e’ R value	P value
Age	-0.431	<0.001	0.330	<0.001
BMI	0.336	<0.001	0.264	0.004
Duration of disease	-0.470	<0.001	0.590	<0.001
HbA1c	-0.395	<0.001	0.256	0.006
HDP	-0.110	0.244	0.108	0.252
DBP	0.100	0.290	-0.153	0.103
CRP	-0.148	0.117	0.063	0.510
CR	0.011	0.904	-0.035	0.711
TG	0.024	0.801	0.099	0.294
TC	-0.025	0.794	0.042	0.654
LDL	-0.143	0.128	0.057	0.545
HDL	0.049	0.611	0.059	0.538
UA	-0.002	0987	-0.015	0.876

BMI, body mass index; SBP, systolic blood pressure; DBP, diastolic blood pressure; SGLT2, Sodium-glucose cotransporter-2: GLP-1, glucagon-like peptide-1; HbA1C, glycated hemoglobin; CPR, C-reactive protein; Cr, creatinine; TC, total cholesterol; TG, triglycerides; HDL-C, high-density lipoprotein cholesterol; UA, uric acid; LDL-C, low-density.

Notably, both E/A and E/e’ values demonstrated significant correlations with key clinical factors, including HbA1C, age, disease duration, and BMI values. However, no significant associations were observed between E/A and E/e’ values and other clinical parameters, such as TG and HBP. Multivariable linear analysis revealed that HbA1C (β= 0.294, p<0.001) and disease duration (β =0.319, p<0.001) could predict reduced E/A (model R2 = 0.381, **Table 4**). Furthermore, HbA1C (β =0.178, p=0.015) and disease duration (β =0.529, p<0.001) were identified as predictor of increased E/e’ (model R2 = 0.436, **Table 5**).

**Table 4:** Multivariate regression analysis for E/A in T2DM patients.

Indicator	Unstandardized β	Standardized β	95%CI	*P* value
Age	-0.008	-0.249	(-0.012, -0.003)	0.002
Duration	-0.015	-0.319	(-0.022, -0.008)	<0.001
HbA1C	-0.061	-0.294	(-0.092, -0.030)	<0.001
BMI	-0.018	-0.195	(-0.033, -0.004)	0.012

BMI, body mass index; HbA1C, glycated hemoglobin.

**Table 5:** Multivariate regression analysis for E/e’ in T2DM patients.

Indicator	Unstandardized β	Standardized β	95%CI	*P* value
Age	0.032	0.166	(0.003, 0.062)	0.029
Duration	0.159	0.529	(0.115, 0.204)	<0.001
HbA1c	0.235	0.178	(0.047, 0.424)	0.015
BMI	0.092	0.154	(0.005, 0.179)	0.037

BMI, body mass index; HbA1C, glycated hemoglobin.

## Discussion

This study depicted that echocardiographic assessment in patients with diabetes revealed diminished E/A values and heightened E/e’ values compared to healthy controls. Subgroup analysis within the diabetic population indicated that patients with a disease duration exceeding 2 years exhibited lower E/A values and higher E/e’ values in comparison to those with a disease duration of 2 years or less. Among patients with a disease duration exceeding 2 years, those with elevated HbA1C levels demonstrated lower E/A values and higher E/e’ values compared to those with lower HbA1C levels. Multivariable linear regression analysis identified HbA1C and diabetes duration as independent risk factors for reduced E/A values and elevated E/e’ values on echocardiography among patients with diabetes, respectively. To accurately evaluate changes in cardiac function in diabetic cardiomyopathy, this study excluded patients with hypertension, coronary heart disease, and valvular disease. Patients with T2DM were matched with healthy individuals based on BMI, gender, age, and smoking status to minimize the impact of these factors on cardiac function (19, 20). The findings underscored impaired diastolic function in patients with T2DM.

In accordance with the latest guidelines from the American Society of Cardiac Ultrasound and the European Society of Cardiovascular Imaging, the assessment of cardiac diastolic function involves parameters measured through Doppler blood flow imaging, including E wave, E’, A wave, and A’. Previous studies have reported various indicators such as prolonged cardiac deceleration time, decreased e’, increased E/e’, reduced E/A, and prolonged isovolumic relaxation time in diabetic patients with normal left ventricular systolic function (21, 22). Additionally, global longitudinal strain of the left ventricle, speckle tracking echocardiography, and strain rate imaging have all confirmed early diastolic dysfunction in patients with diabetes, signifying the apparent progression from diastolic dysfunction to left ventricular systolic dysfunction in diabetic cardiomyopathy (23, 24). Our study substantiates these findings by confirming that diabetic patients with normal ejection fraction exhibit decreased E/A values and increased E/e’ values, highlighting the occurrence of diastolic dysfunction in the early stages of diabetes. Subgroup analysis based on diabetes duration and HbA1C levels further dissected cardiac function. The results indicated that patients with long-standing diabetes and diastolic dysfunction experienced worse outcomes compared to patients with initial-onset diabetes but no systolic dysfunction. Correlation analysis revealed a negative association between the E/A parameter and disease duration, while E/e’ exhibited a positive correlation with disease duration. These findings suggest that diabetic cardiomyopathy gradually develops and progresses with the duration of diabetes, while no changes in cardiac function were observed in patients with initial-onset diabetes. Advanced cardiac magnetic resonance imaging (CMRI) technology, as explored by Japanese researchers, has provided insights into cardiac conditions in young patients with T2DM (under 40 years of age). Their findings revealed a close relationship between early diastolic dysfunction and the duration of diabetes (25). Furthermore, Shah et al. observed geometric changes in cardiac remodeling and significant reductions in myocardial diastolic function in obese adolescents with T2DM, suggesting an increased risk of early heart failure in this population (26). CMRI studies have consistently demonstrated lower early diastolic peak strain rate in young individuals with T2DM (18-40 years old) compared to normal subjects, indicating the presence of subclinical diastolic dysfunction in the early stages of diabetes and a susceptibility to heart failure (27), aligning with the observations from our study.

Persistent hyperglycemia can induce diastolic dysfunction through various mechanisms, including abnormal glucose and lipid metabolism, inflammation, oxidative stress, activation of the renin-angiotensin-aldosterone system (RAAS), and myocardial microvasculopathy. These processes lead to cardiomyocyte apoptosis, hypertrophy, and fibrosis (5, 28). The severity of myocardial diastolic dysfunction is notably higher in patients with poorly controlled blood glucose levels, HbA1C levels surpassing 7.5%, and extended disease durations. Correlation analysis demonstrated a negative association between HbA1C and the diastolic function parameter E/A, while a positive correlation was found between HbA1C and E/e’. Increased HbA1C levels and of glucose and lipid metabolism contribute to cardiac dysfunction. Meta-analyses consistently indicate a significant association between higher HbA1C levels and an increased incidence of congestive heart failure (27). Recent studies have identified HbA1C as an independent predictor of left ventricular myocardial deformation and tissue abnormalities in patients with T2DM and preserved ejection fraction (29). However, Shun Yokota suggested that left ventricular diastolic function is more strongly associated with hyperglycemic variability rather than HbA1C levels in T2DM (30). Larger patient cohorts are necessary for further investigation to validate these differences. Consistent with our findings, obesity and smoking in adolescents have been linked to increased left ventricular volume and reduced myocardial diastolic function (20, 31, 32). Our study also depicted the impact of BMI on cardiac function, but no significant correlation was seen between smoking and cardiac function parameters, potentially due to the limited number of smokers included in our study. Importantly, our results highlighted age as a key clinical factor significantly correlated with T2DM. Nearly half of people with diabetes are middle-aged and elderly, making this group particularly vulnerable (33). Factors such as age and T2DM contribute to increased frailty in this population (34, 35). Furthermore, insulin resistance is prevalent among frail elderly individuals, adding complexity to the clinical treatment of elderly patients with diabetes and heart failure (36, 37). Urgent attention is required for the development of treatment programs and antidiabetic drugs that can effectively reduce glucose levels and provide cardiac protection. Several studies have explored these areas (38, 39), which will be a focus of our future research. Admittedly, this study has several limitations that should be acknowledged: Firstly, the study is constrained by a short follow-up period and a narrow window for the cross-sectional study, potential introducing observation bias. Secondly, there is an inadequacy of research regarding the potential enhancement of diastolic dysfunction through antidiabetic medications. Thirdly, insufficient attention has been given to dietary control and the impact of other blood sugar management approaches, such as exercise.

## Conclusions

This study offers compelling evidence of early diastolic dysfunction in diabetic cardiomyopathy, observable in patients with preserved ejection fraction during the early stages of diabetes. Moreover, cardiac diastolic function demonstrated significant associations with diabetes duration and HbA1C levels. This emphasizes the importance of early echocardiography assessment and screening of E/A and E/e’ in newly diagnosed diabetes patients for the prompt detection and monitoring of diabetic cardiomyopathy progression.

## Data availability statement

The raw data supporting the conclusions of this article will be made available by the authors, without undue reservation.

## Ethics statement

The protocol has been reviewed and approved by the Ethics Committee of the Second Affiliated Hospital of Hebei Medical University. The studies were conducted in accordance with the local legislation and institutional requirements. The participants provided their written informed consent to participate in this study.

## Author contributions

NL: Conceptualization, Writing – original draft. MZ: Data curation, Formal analysis, Writing – review & editing. LY: Data curation, Formal analysis, Writing – review & editing. YC: Data curation, Formal analysis, Writing – review & editing. HZ: Conceptualization, Writing – review & editing.
